# Use of a Custom-Made Patellar Groove Replacement in an American Staffordshire Terrier Puppy with a Severe Bone Defect in the Femoral Trochlea Caused by Hematogenous Osteomyelitis

**DOI:** 10.3390/ani14060909

**Published:** 2024-03-15

**Authors:** Enrico Panichi, Sara Sassaroli, Giorgio Maria Ciccarese, Valentina Riccio, Caterina Balestriere, Marco Barbaccia, Fulvio Cappellari, Ekaterina Burkhan, Angela Palumbo Piccionello

**Affiliations:** 1 Orthopaedic Veterinary Trauma Center (CTO VET), Via C. Festa 9, 16011 Arenzano, Italy; enricopanichi76@gmail.com (E.P.); ciccaresegiorgio@gmail.com (G.M.C.); caterinabalestriere@gmail.com (C.B.); m.barbaccia89@gmail.com (M.B.); fulvio.cappellari@ctovet.com (F.C.); 2 School of Biosciences and Veterinary Medicine, University of Camerino, Via Circonvallazione 93/95, 62024 Matelica, Italy; valentina.riccio.dvm@gmail.com (V.R.); angela.palumbo@unicam.it (A.P.P.); 3 Bonabyte, Begovoy Proezd 7, 125284 Moscow, Russia; k.burkhan@bonabyte.net

**Keywords:** osteomyelitis, custom-made patellar groove replacement, femoral trochlea, 3D-printing, patellar luxation, stifle joint, dog

## Abstract

**Simple Summary:**

Osteomyelitis is a bone infection disease causing progressive inflammation. Bone lysis, periosteal reactions and ischemic regions of infected necrotic and devitalized tissue are likely secondary abnormalities. A prolonged antibiotic therapy, an abundant lavage of the affected region and several revision surgeries to debride infected and necrotic bone are paramount treatments for osteomyelitis. The treatment of extensive bone defects and functional damage may require the use of prosthetic surgery, allowing the anatomical and functional recovery of the affected area, and in some cases, it is necessary to use a customized prosthesis for a better anatomical and functional adaptation. In veterinary medicine, the implementation of 3D-printing technologies and the application of custom-made surgical implants and prosthetics have enabled the management of intricate orthopedic conditions that were previously only addressed through salvage procedures, such as arthrodesis or amputations. The purpose of this report is to describe the surgical technique and outcome of a custom-made patellar groove replacement in a puppy with a severe bone defect in the femoral trochlea caused by hematogenous osteomyelitis. This surgery showed excellent short-, medium- and long-term outcomes, and it is the first report on a custom-made patellar groove replacement available in the literature.

**Abstract:**

An 8-month-old male American Staffordshire terrier was referred for a no-weightbearing lameness of the right pelvic limb, hyperthermia, lethargy and inappetence. Two months before, endocarditis was diagnosed and treated in another veterinary hospital. Orthopedic, radiographic and tomographic examinations revealed a bone sequestrum of 4 × 1.4 cm and active periosteal reaction of the caudo-lateral cortical in the metaphysis and the distal third of the right femoral diaphysis, medullary osteolysis and interruption of the cranio-medial cortical profile, with involvement of the femoral trochlea leading to a secondary medial patella luxation. Hematogenous osteomyelitis was the suspected diagnosis. Once skeletally mature, after 4 months from surgical debridement and aggressive antibiotic therapy against Klebsiella oxytoca revealed by a bacteriological exam, the patient underwent prosthetic surgery for the application of a custom-made patellar groove replacement (PGR) to fill the bone defect and restore the femoral trochlea surface. Despite the serious injury that afflicted the right pelvic limb, the surgery had satisfactory outcomes until the last 18-month postoperative follow up.

## 1. Introduction

Osteomyelitis is an inflammatory condition of the bone and bone marrow caused by an infectious agent such as bacteria, virus or fungi [1,2]. Osteomyelitis has been classified based on the pathway through which the infectious agent enters the site of infection: hematogenous and post-traumatic or direct inoculation [1]. Despite bones being naturally resistant to colonization and infection in physiologic conditions, post-traumatic osteomyelitis, the most common source of osteomyelitis in dogs and cats, can develop as a result of a penetrating wound (e.g., foreign body or bite wound) or the introduction of foreign material (e.g., implants or prosthesis) [3,4,5,6]. A less common condition is hematogenous osteomyelitis, which usually affects young animals and animals with abnormal immune systems and results from the spread of bacteria, localized to a distant site, to the bone via the bloodstream. Due to anatomic differences in the macro- and microcirculation (capillaries with an incomplete basement membrane and gaps between endothelial cells, and a sluggish blood flow within the metaphyseal capillaries), it most commonly affects the metaphyseal regions of long bones [1,6,7,8,9].

A patient suffering from osteomyelitis on a long bone segment usually shows lameness in the affected limb, localized soft tissue swelling and pain. Muscle atrophy and draining tracts may be present. In addition, the affected animals are often systematically ill and clinical signs can include fever, inappetence and lethargy. Bone resorption, bone lysis, periosteal reactions and ischemic regions of infected necrotic and devitalized tissue can be detected [1,10].

In the case of osteomyelitis, immediate and aggressive therapy is necessary [1]. The resolutive treatment of osteomyelitis sometimes requires a long hospital recovery, a prolonged antibiotic therapy, an abundant lavage of the affected region and the debridement of infected and necrotic bone [6,11]. Nevertheless, the resulting bone loss and significant defects may lead to a high risk of protracted bone healing times and of infection recurrence [6]. Amputation is the last and unwanted therapeutic option for the intractable osteomyelitis of a single limb [1,6].

The treatment of extensive bone defects and functional damage may require the use of prosthetic surgery, allowing the anatomical and functional recovery of the affected area [12]. Similar to human medicine, the veterinary field also offers a range of standard-sized joint prostheses that are readily available on the market. Total hip replacement (THR), total knee replacement (TKR), patellar groove replacement (PGR) and total elbow replacement (TER), but also compartmental prostheses such as biomechanically anatomic non-constrained compartmental partial elbow replacement (BANC PER), are some of the prostheses available [13,14,15,16]. Despite the different prosthetic models and sizes, in some cases, the use of customized prosthesis with specific characteristics is necessary for a better anatomical and functional adaptation [12,17,18]. In the last few years, in the human and veterinary orthopedics fields, the use of three-dimensional (3D) printing has been found to be a valuable method for fabricating bespoke implants tailored to the precise requirements of recipients [12,17]. In veterinary medicine, the application of custom-made surgical implants and prosthetics has proven indispensable for addressing intricate orthopedic conditions and revision surgeries, such as nonunion or malunion fractures, tumors, bone deformities, severe traumatic injuries and substantial bone loss in the absence of infection [12].

The objective of this case report is to describe the surgical technique and outcome of a custom-made PGR in an American Staffordshire terrier puppy with a severe bone defect in the femoral trochlea caused by hematogenous osteomyelitis. In our knowledge, this is the first report on custom-made PGR available in the literature.

## 2. Case Report

An 8-month-old American Staffordshire terrier weighing 25 kg was referred to the Orthopedic Veterinary Trauma Center (Arenzano, Italy) for no-weightbearing lameness of the right hind limb. The lameness had suddenly arisen two weeks earlier without any traumatic episode, and it showed a progressive worsening. During this time, the owner reported that the dog experienced recurrent episodes of intermittent hyperthermia. Two months before, endocarditis was diagnosed in the patient and treated with antibiotic therapy in another veterinary hospital.

The rectal temperature was increased (40.5 °C). The patient manifested lethargy, inappetence and anorexia. A complete blood count and the serum biochemical profile revealed leukocytosis (27.10 × 109/L; reference range: 6.00–17.00 × 109/L) and an increment in total serum protein (8.3 g/dL; reference range: 5.2–8.2 g/gL) and hyperglobulinemia (5.2 g/dL; reference range: 2.5–4.5 g/gL).

**Figure 1:**
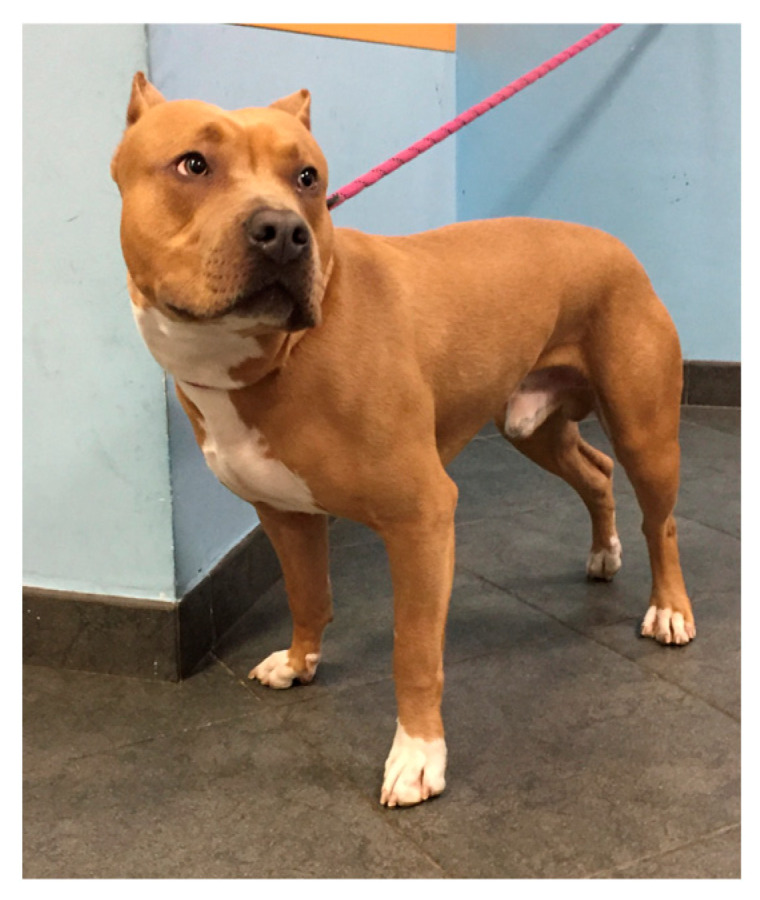
The 8-month-old American Staffordshire terrier referred to the Orthopaedic Veterinary Trauma Center (Arenzano, Italy) for no-weightbearing lameness of the right hind limb.

**Figure 2:**
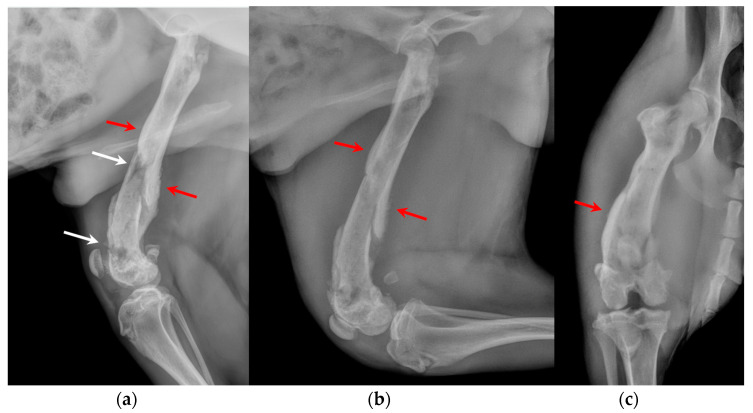
(**a**) Extended medio-lateral, (**b**) flexed medio-lateral and (**c**) cranio-caudal radiographic projections of the right stifle joint and femoral diaphysis, performed by reference colleagues, highlighted an extensive periosteal reaction of the diaphysis and femoral metaphysis (red arrows) and a radiolucent area (white arrows) in the distal metaphysis.

The orthopedic examination revealed no-weightbearing lameness (grade IV on a scale from 0 to IV) [19] and severe edema of the right hind limb, pain on manipulation of the right stifle, joint swelling and grade III medial patellar luxation (MPL) [20] (Figure 1, Video S1). Caudo-cranial and medio-lateral radiographic projections of the right femur, performed by reference colleagues, highlighted an extensive periosteal reaction of the diaphysis and femoral metaphysis, and a radiolucent area in the distal metaphysis (Figure 2).

**Figure 3:**
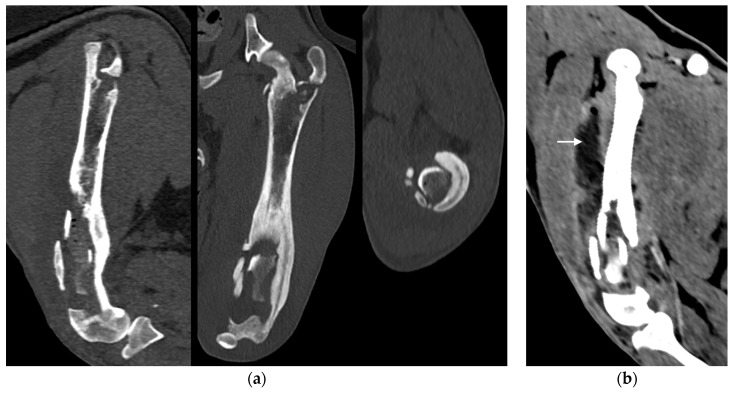
(**a**) Sagittal, frontal and transverse tomographic images of the metaphysis and the distal-middle third of the femoral diaphysis revealed medullary osteolysis, a wide interruption of the cranio-medial cortical profile with corticomedullar fragment of 4 × 1.4 cm (length × width) medially dislocated at the level of the medullary cavity (bone sequestrum) and an extensive periosteal reaction of the caudo-lateral cortex of the femoral distal diaphysis. (**b**) Widespread subcutaneous and perifascial abscess lesions were also reported (white arrow).

Under anesthesia, a computed tomography study (CT; Somatom Emotion 16, Siemens, Munich, Germany) of the hind limbs was performed. The tomographic images revealed medullary osteolysis of the metaphysis and the distal-middle third of the femoral diaphysis. In the metaphysis and the distal third of the femoral diaphysis, a bone sequestrum was found: a wide interruption of the cranio-medial cortical profile, with involvement of the femoral trochlea, was observed with corticomedullar fragment of 4 × 1.4 cm (length × width) medially dislocated at the level of the medullary cavity; the fragment was surrounded by a large hypodense halo. Secondary MPL and widespread subcutaneous and perifascial abscess lesions were also reported (Figure 3).

The patient was subsequently subjected to arthrocentesis for bacteriological and cytological examination of synovial fluid. It appeared cloudy and similarly purulent at the macroscopic vision. During the same session, a joint irrigation with a sterile physiological solution was performed as reported in literature [21].

Based on the anamnestic and clinical data, on radiographic and tomographic examination and on macroscopic appearance of synovial fluid, the diagnostic suspicion was septic arthritis of the right stifle joint and osteomyelitis of right distal femur.

Consequently, the patient was hospitalized in order to provide intravenous (IV) broad-spectrum antibiotic therapy (amoxicillin and clavulanic acid 20 mg/kg IV twice daily), anti-inflammatory therapy (meloxicam 0.1 mg/kg SC once a day) and maintenance fluid therapy (Ringer’s lactate 2 mL/kg/h IV), pending the results of bacteriological and cytologic examination.

After 5 days, the results of cytology and bacteriological exams of the synovial fluid showed a severe inflammatory process and the presence of Klebsiella oxytoca, a multidrug-resistant bacterium which was sensitive to the antibiotic nitrofurantoin. These results and the images obtained from tomographic scans confirmed the diagnosis of osteomyelitis and septic arthritis.

**Figure 4:**
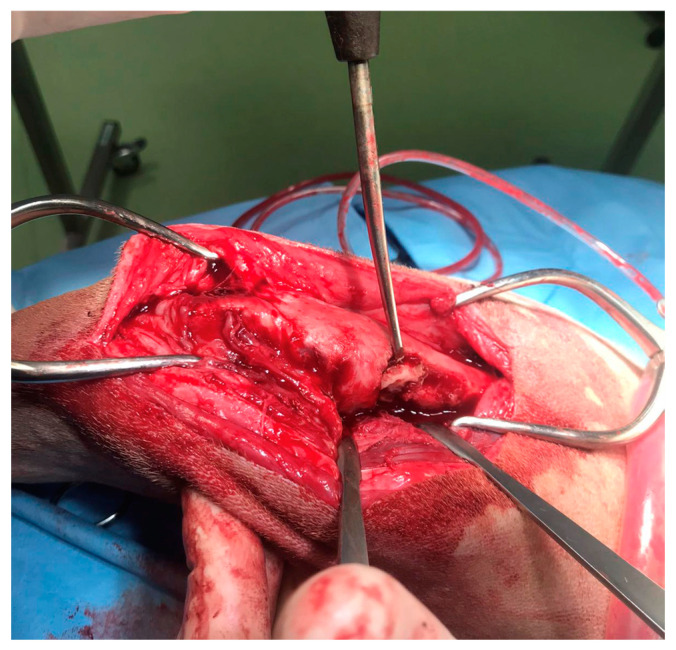
The intraoperative image of the bone sequestrum and the surgical debridement of the necrotic tissue.

In agreement with the owner, a surgical procedure was performed in order to remove the bone sequestrum, debride necrotic tissues and perform a copious lavage of the affected region (Figure 4). A modified Robert Jones splint was applied for 48 h after the surgery. The patient was hospitalized for 48 h and then discharged with a specific antibiotic therapy (nitrofurantoin 4 mg/kg PO twice daily) and anti-inflammatory therapy (meloxicam 0.1 mg/kg PO once daily). The antibiotic and anti-inflammatory therapy was prescribed and administered to the patient for 30 days once the patient was discharged.

At 12 months of age, once skeletally mature, the patient was again subjected to an orthopedic and radiographic evaluation and CT scans of the pelvis and the hind limbs. Orthopedic examination revealed a grade III lameness (on a scale from 0 to IV) [19], considerable muscle hypotrophy, pain on manipulation of the right stifle, joint swelling and grade III MPL [20]. The severe edema of the limb observed during the first orthopedic evaluation disappeared (Video S2).

The CT showed an extensive bone defect on the metaphysis and on the distal third of the femoral diaphysis, with loss of the cranio-medial cortical of the distal femur and of the femoral trochlea and resulting medial patella luxation of the right hind limb.

A 3D reconstruction of the hind limbs was performed in order to evaluate their alignment and to design a custom-made PGR: a custom-made implant that fills the bone gap and allows the restoration of the articular surface of the missing femoral trochlea. BonaPlanner computer-aided design CAD software (Bonabyte LLC, Moscow) was used to perform the 3D reconstruction of the hind limbs and the design and manufacture of the cutting guide and custom-made PGR. After uploading the DICOM files of the CT images onto BonaPlanner software, the DICOM files was segmented to STL files to obtain 3D models and allow the imaging processing.

**Figure 5:**
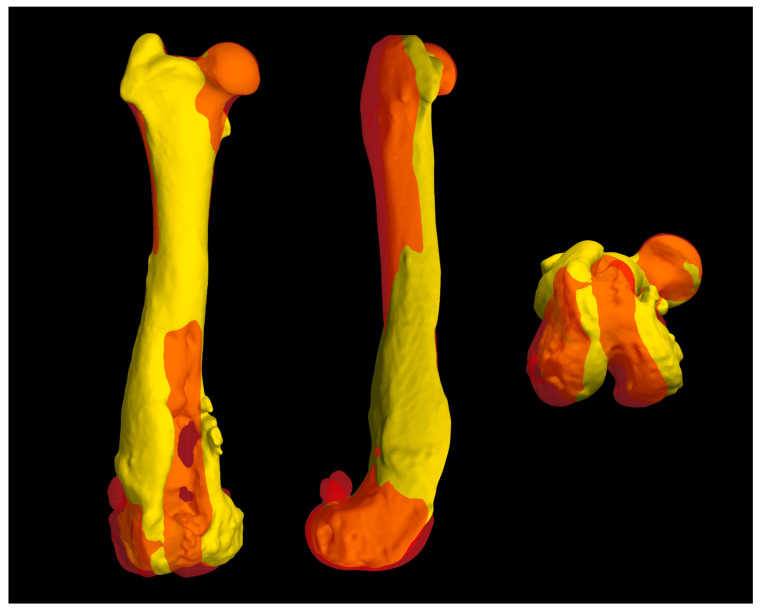
The superimposition of normal and pathological femur measurements obtained using BonaPlanner computer-aided design CAD software (Bonabyte LLC, Moscow, Russia) allowed to assess the presence of deformities in the frontal, sagittal and axial planes, comparing the pathological right hindlimb and the contralateral hindlimb. The superimposition of the 3D reconstructions showed the presence of a slight distal femur varus of the right hindlimb, which did not require surgical correction.

The 3D models of normal and pathological femurs were made using the same software and their superimposition allowed us to observe the presence of a slight distal femur varus of the right hindlimb, so slight as to not require surgical correction. No other differences were detected in the frontal, sagittal and axial planes (Figure 5). Subsequently, the custom-made PGR and the osteotomy guide were designed in order to perform a prosthetic surgery to restore the articular surface of the femoral trochlea and resolve the bone defect.

### 2.1. Planning of the Osteotomy Guide

An osteotomy guide was created to prepare the site of the prosthetic implant. The guide was designed in order to reproduce the osteotomy proposed by Dokic et al. for the removal of the femoral trochlear for the application of Kyon^®^ PGR (KYON AG, Zurich, Switzerland) [16]. The authors suggested to perform an osteotomy from the level of the origin of the tendon of the long digital extensor muscle (extensor digitorum pedis longus, EDPL) to the proximal end of the trochlea [16]. The osteotomy guide accurately matched the cranial surface of the distal femoral epiphysis, in particular the partially intact lateral trochlear ridge, which represented an important and easily recognizable landmark during surgery for the correct application of the osteotomy guide on the bone. In the guide, there were four holes that allowed the insertion of four 2.0 mm Steinmann pins in order to fix the guide to the bone during osteotomy execution, and two slots, laterally and medially, that permitted the entry and exit of a saw blade 1 mm thick (DePuy Synthes Vet, West Chester, PA, USA). The four holes for pins’ application were designed to match the holes for the screws of the custom-made implant. The osteotomy guide was made of Nextdent SG (Surgical Guide) by 3D SYSTEM^®^ and printed with a 3D printing machine (Form 2, Formlabs, MA, USA).

### 2.2. Planning of the Custom Trochlear Prosthesis

**Figure 6:**
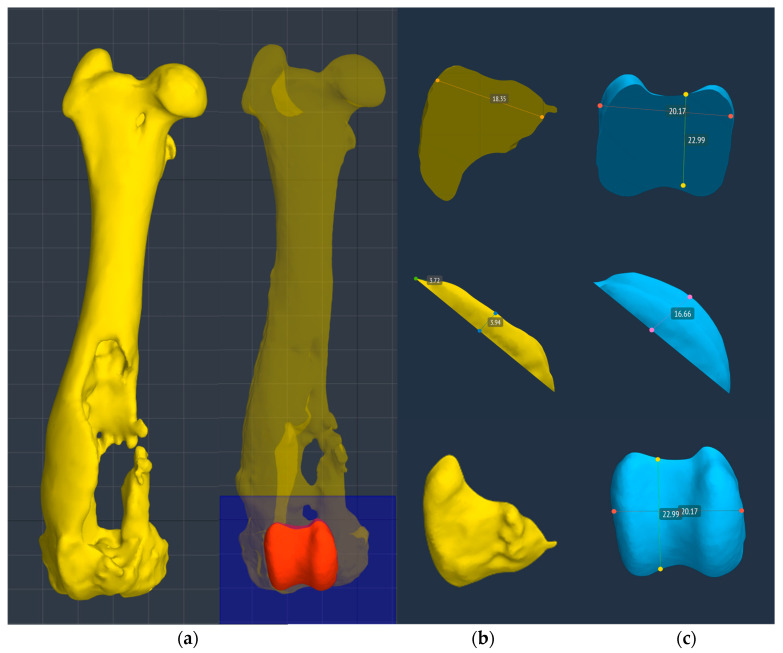
(**a**) The surface of the prosthesis that reproduced the femoral trochlea perfectly replicated the contralateral trochlea groove (red trochlea groove) and it was planned using BonaPlanner computer-aided design CAD software (Bonabyte LLC, Moscow, Russia). Reconstruction of the cranial surface (**b**) of the pathological distal femoral epiphysis with the partially intact lateral trochlear ridge and (**c**) of the contralateral distal femoral epiphysis, in particular of the femoral trochlea.

**Figure 7:**
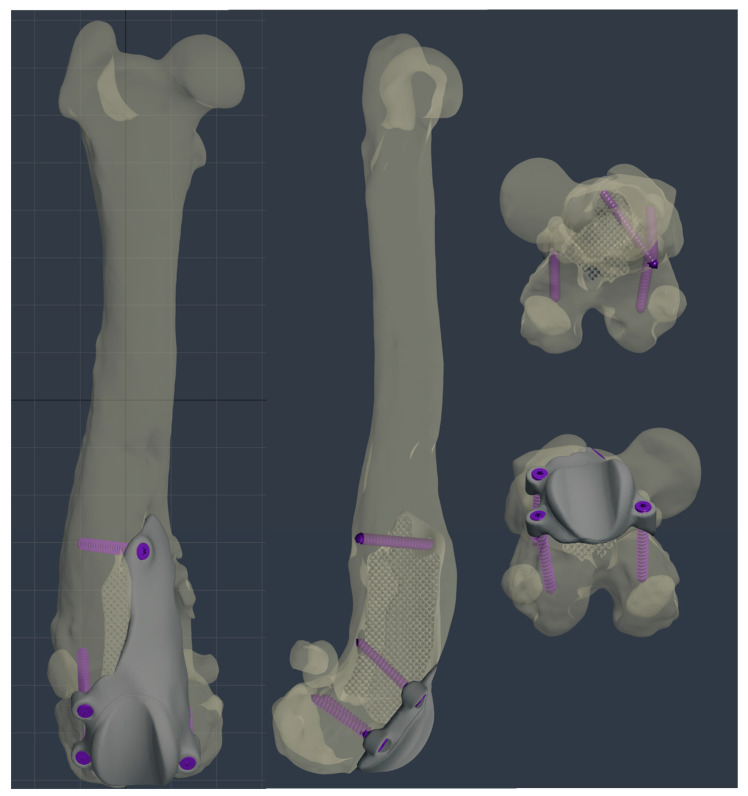
The custom-made PGR prosthesis was planned using BonaPlanner computer-aided design CAD software (Bonabyte LLC, Moscow, Russia). The prosthesis filled the bone defect and anatomically reproduced the shape of the femoral trochlea on the upper face of the implant. Four holes for screws was designed one lateral and one medial to the distal margin of the lateral and medial trochlear ridges, one lateral to the proximal end of the lateral trochlear ridge and one in the center of the femoral diaphysis, in the most proximal end of the bone defect.

**Figure 8:**
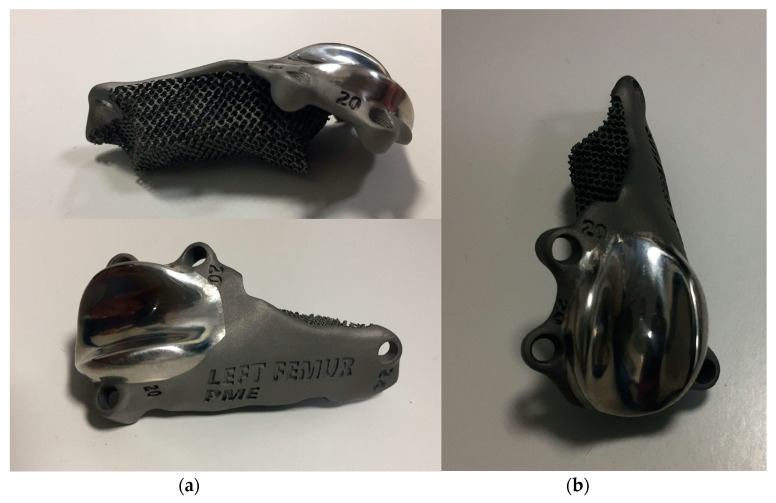
Photographs of (**a**) lateral (**above**), cranio-medial (**below**) and (**b**) cranial aspect of the custom-made PGR prosthesis. The implant was made of titanium Ti6Al4V ELI (Grade 23) and the mirror surface of the prosthesis that reproduces the femoral trochlea was made by polishing with a diamond base abrasive material made by hand. The portion of the prosthesis designed to fill the bone defect was completely porous to promote the bone growth and osteointegration.

The custom-made PGR prosthesis was made of titanium Ti-6Al-4V extra low interstitial (ELI) (Grade 23) and was printed with a GE^®^ M2 Series 5 laser. The prosthesis was designed in order to fill the bone defect and anatomically reproduce the shape of the femoral trochlea on the upper face of the implant. The mirror surface of the prosthesis that reproduces the femoral trochlea was made by polishing with a diamond base abrasive material made by hand to reduce the coefficient of friction, similar to the prosthetic component of Kyon^®^ PGR [16]. The portion of the prosthesis designed to fill the bone defect had a porous structure with a cell size of 0.7–1 mm to promote the bone growth and osseointegration. The custom-made PGR prosthesis was secured to the bone with four locking screws (2.7 mm, Ti-6Al-4V ELI). The four holes for screws were designed one lateral and one medial to the distal margin of the lateral and medial trochlear ridges, respectively; one lateral to the proximal end of the lateral trochlear ridge and one in the center of the femoral diaphysis, in the most proximal end of the bone defect (Figure 6, Figure 7 and Figure 8).

### 2.3. Surgery

The dog was premedicated with methadone (0.2 mg/kg intramuscularly (IM)) and dexmedetomidine (1 µg/kg IM). General anesthesia was induced with propofol (2 mg/kg IV) and maintained with isoflurane in oxygen after endotracheal intubation. A loco-regional block with ropivacaine (1 mg/kg) of the sciatic and femoral nerves of the right hind limb was performed and constant rate infusion of fentanyl (5–20 µg/kg/h) was applied to provide analgesia. The antibiotic cefazolin (22 mg/kg IV) was administered one hour before surgery and repeated every 90 min until the end of the surgery.

The affected limb was aseptically prepared for surgery from the metatarsal–phalangeal joints to the coxo-femoral joint. The patient was positioned in dorsal recumbency and a lateral parapatellar approach was performed [22], prolonging the incision to the proximal end of the bone defect.

**Figure 9:**
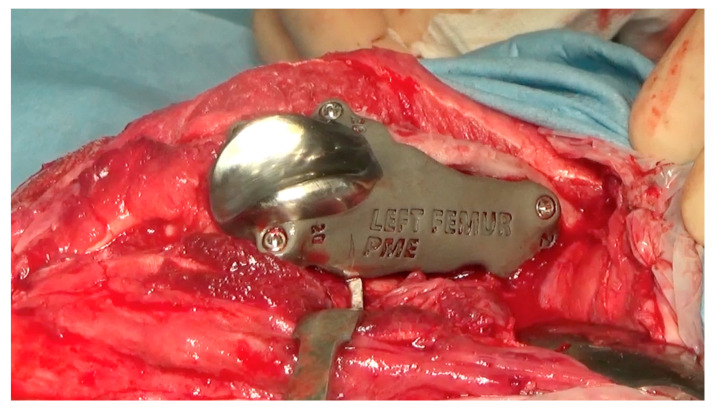
The intraoperative image of the custom-made PGR stabilized to the bone.

After reflection of the patella and the exposure of the cranial surface of the distal femur, the debridement of the fibrous tissues from the cranial surface of the distal femoral metaphysis was performed using a pneumatic drill with diamond burs (Surgairtome two, Hall), in order to allow the placement of the osteotomy guide. Once the guide was properly positioned, the guide was fixed with four 2.0 mm Steinmann pins into the four designed holes. The osteotomy was performed with a motorized oscillating saw inserting the saw blade of 1 mm into the lateral slot in order to perform a lateral–medial osteotomy: the plan of osteotomy extended from the level of the origin of the tendon of the EDPL to the proximal end of the partially intact lateral trochlear ridge. The osteotomy guide was removed and the custom-made PGR prosthesis was applied. Once it was verified that the holes made for the application of the pins fit perfectly with the holes of the implant for the insertion of the screws, the custom-made PGR was stabilized to the bone with the four 2.7 mm titanium locking screws described previously (Figure 9).

An intraoperative swap from the surgical site was performed for a bacteriological examination. The patella was reduced and tested in flexion and extension and an abundant lavage of the surgical field was performed. Capsulorraphy was executed with interrupted absorbable sutures and the subcutaneous tissues and skin were closed routinely.

### 2.4. Postoperative Evaluation and Management

**Figure 10:**
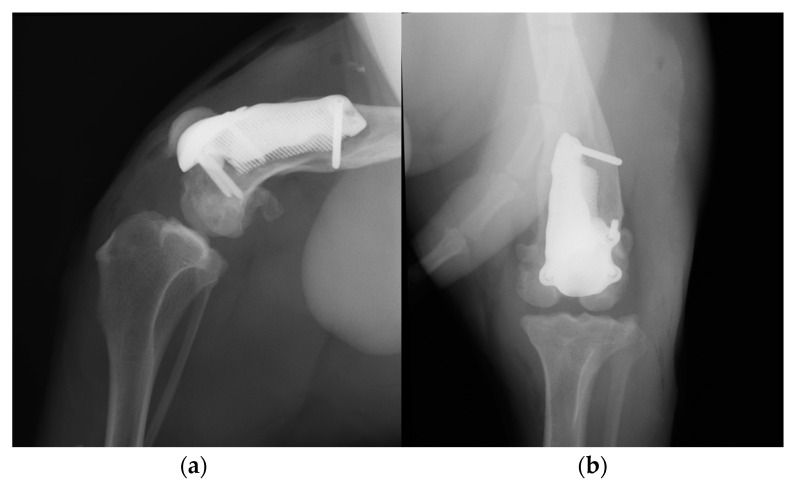
Postoperative medio-lateral (**a**) and caudo-cranial (**b**) radiographs of the stifle joint showing the appropriate prosthesis position, the correct length of the screws and the reduction in the patella.

Postoperative medio-lateral and caudo-cranial radiographs of the stifle joint were performed to evaluate the proper prosthesis position and the length of the screws and to confirm the patellar reduction (Figure 10). To improve the postoperative assessment, CT scans of the right stifle joint were obtained. A modified Robert Jones splint was applied for 24 h postoperatively. Meloxicam (0.1 mg/kg) once a day and amoxicillin and clavulanic acid (20 mg/kg) twice a day were prescribed for 10 days, pending the results of a bacteriological examination, and only short walks on the leash to be gradually increased during the two months of postoperative rehabilitation were recommended. After 5 days, the bacteriological exam results were negative, and the antibiotic therapy was continued as indicated previously.

A grade III lameness (on a scale from 0 to IV) [19] of the right hind limb was observed 24 h postoperatively. Crepitus, mild pain and swelling of the right stifle joint were detected on manipulation, and the range of motion (ROM) was decreased due to the limited flexion and extension (Video S3a). The estimated angle of extension and flexion of the right stifle was 150–110°, respectively, and the thigh circumference was 28 cm.

Daily physiotherapy was started one day after the surgery. Massage of the hind limb and passive movement of the right stifle joint, associated with instrumental therapies such as laser and tecar, were performed for 30 days in order to reduce inflammation and improve the joint excursion. Later, the active mobilization of the joint was also stimulated by an obstacle course, and physiotherapy was performed once a week for 6 months.

**Figure 11:**
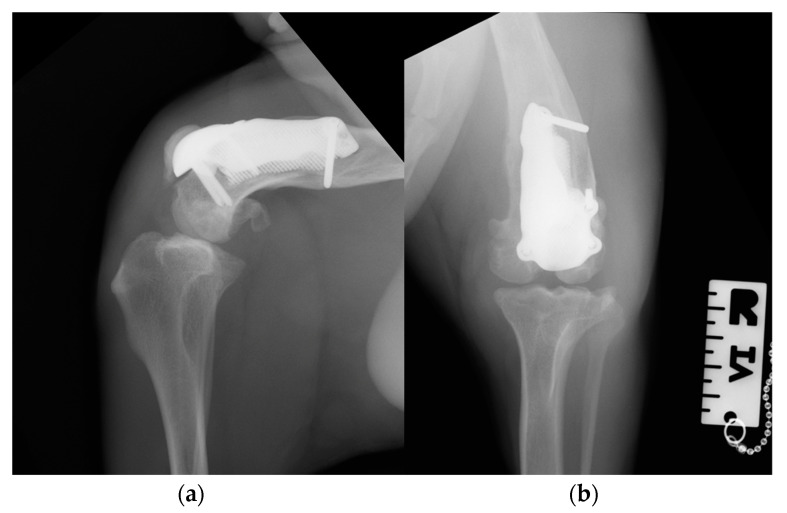
Three-month postoperative medio-lateral (**a**) and caudo-cranial (**b**) radiographs of the stifle joint showing the absence of implant-associated complications, such as implant loosening and migration, a thickening of the patellar ligament and mild osteoarthritic changes in the distal pole of the patella compared to postoperative radiographs.

At the 3-month postoperative follow-up, the orthopedic, radiographic and tomographic examination were repeated. The patient showed a grade II right hind limb lameness (on a scale from 0 to IV) [19] (Video S3b). On stifle joint palpation and manipulation, crepitus persisted. The arthralgia and capsular ectasia decreased compared to the postoperative follow-up, and mild pain was elicited upon palpation of the patellar ligament. The estimated angle of extension and flexion of the right stifle was 150–90°, respectively. The radiographic and tomographic evaluations revealed the absence of implant-associated complications, such as implant loosening and migration, a thickening of the patellar ligament, signs of suspicious desmitis and mild osteoarthritic progression at the distal pole of the patella (Figure 11).

**Figure 12:**
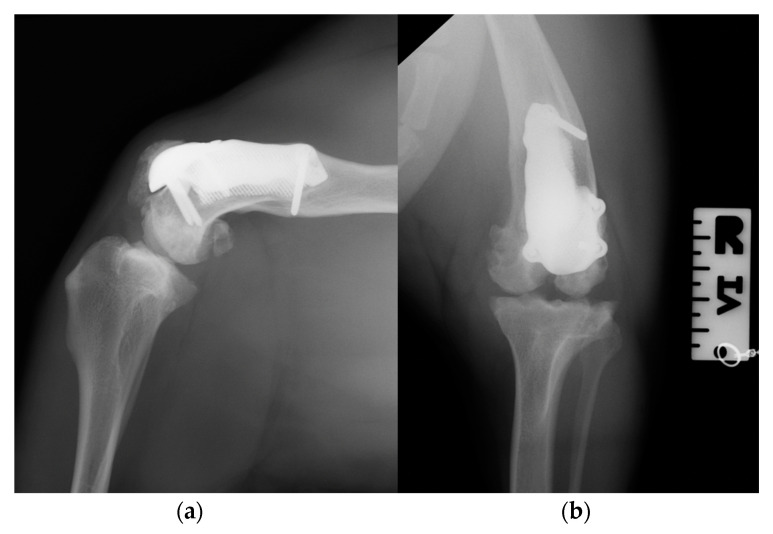
Twelve-month postoperative medio-lateral (**a**) and caudo-cranial (**b**) radiographs of stifle joint showing a good osteointegration of the implant and osteoarthritic changes in the distal pole of the patella compared to postoperative and 3-month postoperative radiographs.

At the 6-, 12- and 18-month follow-up (Video S3c), the orthopedic evaluation revealed crepitus, but a grade 0 right limb lameness and absence of patellar ligament desmitis and palpation of the right stifle joint did not elicit pain. The estimated angle of extension and flexion of the right stifle was 160–80°, respectively, while the ROM of the contralateral healthy limb was 160° in extension and 40° in flexion. The thigh circumference of the pathologic limb was 40 cm compared with 47 cm of the healthy one. The radiographic images showed a good osseointegration of the implant and progression of degenerative changes in the distal pole of the patella (Figure 12).

## 3. Discussion

This report describes the use of a custom-made PGR prothesis to treat an extensive bone defect and to restore the articular surface of the femoral trochlea in an American Staffordshire terrier puppy affected by a severe hematogenous osteomyelitis of the metaphysis and of the distal-middle third of the right femoral diaphysis.

To our knowledge, this is the first report on a custom-made PGR available in the literature [12,16,23]. Despite the serious injury that afflicted the right pelvic limb, the surgery had satisfactory outcomes until the last 18-month postoperative follow-up. The patient fully resumed the functionality of the pelvic limb and no major complications were detected [24]. A complication detected was the patellar ligament desmitis revealed at the 3-month postoperative follow-up. This minor complication was managed by controlled physical exercise and physiotherapy, without the administration of additional drugs [24]. This complication was expected because for four months (time elapsed from the first visit at 8 months of age to the prosthetic surgery at 12 months of age) the soft tissues adapted to a deformed and incomplete femoral trochlea leading to the failure of the stifle extensor mechanism [25]. The application of the custom-made PGR prosthesis and the reduction in the patella determined a cranial translation of the patella, resulting in an increase in the strain on the patellar ligament, which is a cause of the development of ligament desmitis [26]. In addition, during postoperative follow-ups, the crepitus persisted and osteoarthritic changes in the distal pole of the patella were observed. The cranial translation of the patella could cause an increase in retropatellar pressure, which could be responsible for the degenerative processes [27].

The complete resorption of the cranio-medial cortex of the distal femur with the involvement of the femoral trochlea and the presence of a bone defect represent a complex orthopedic condition to face. The use of a standard PGR to restore the femoral trochlea surface was precluded because of the loss of the bone surface to apply the implant [16], so it was decided to design a custom-made prosthesis using 3D-printing technologies. The implementation of these innovative surgical techniques has enabled the management of highly complex situations that were previously only addressed through salvage procedures, such as arthrodesis or amputations [12]. In veterinary practice, despite the growing interest, the application of 3D-printing technologies is in the early stage [28,29,30]. To date, 3D printing in veterinary orthopedics is used for the manufacturing of anatomical models (for preoperative planning and surgical rehearsal, education, communication), of medical devices (such as custom-made plate, saw or drill guide, implants and prosthesis) and in tissue engineering (such as custom-designed scaffold and surface coating for osseointegration [12,31,32,33].

The custom-made PGR had several advantages. Firstly, the component of the prosthesis designed to fill the bone defect was completely porous for long-term biologic fixation, promoting bone growth and osseointegration [16,31]. Secondly, the prosthesis completely removes the bone defect, which could be a site of bacterial colonization resulting in recurrent infections [6]. Thirdly, the upper face of the implant anatomically reproduces the shape of the contralateral femoral trochlea, allowing the repositioning of the patella, preventing its dislocation and restoring the function of the patella–femoral joint. In addition, the surface of the prosthesis that reproduces the femoral trochlea was treated with an amorphous diamond base coating to reduce the coefficient of friction [16]. As a last advantage, but no less important, four holes for screws were designed to secure the custom-made PGR to the bone despite the alteration of the anatomy of the distal femur. The rationale for the screw position was to bridge the defect and to fix the prosthesis immediately.

The custom-made PGR was manufactured with the titanium alloy, a common alloy used in biomedical components, highly biocompatible and corrosion resistant like pure titanium but with a superior fatigue resistance [34,35]. In particular we used Ti-6Al-4V ELI, an alloy where the interstitial elements such as oxygen, carbon and iron are deliberately kept low in order to improve the fracture toughness and ductility [36].

The high cost of the implant and the complexity of the manufacturing are disadvantages of the use of a custom-made PGR. The planning and implementation of the implant is time-consuming, and the use of appropriate software and the communication with experts in the field of CAD design are necessary. In addition, the prosthetic surgery was performed once the dog had completed skeletal growth because custom-made implants are not suitable for growing dogs: the prosthesis does not subsequently adapt to the growth of the patient, leading to possible implant failure. Another disadvantage of a custom-made implant is that usually requires the realization of cutting guides where it is necessary to perform osteotomies for the placement of the prosthesis [37]. The advantage of the custom-made cutting guides is the surgical precision improvement [12], but they require a proper fit on the bone structure and a precise and stable placement [37,38,39]. In addition, they increase the production costs and the planning time. In our report, a major point for the correct execution of the surgery was in fact the debridement of the fibrous tissues from the cranial surface of the distal femoral metaphysis, in order to allow the proper placement of the osteotomy guide, and the fixation of the guide with Steinmann pins. The fibrous tissues and adhesions that developed during these months were abundant and difficult to remove, so it was decided to use a high speed bur and a surgical electric cutter to obtain a better debridement. For surgical application of the PGR that was commercially available, the cut of the trochlea is performed in a free-hand fashion following anatomical landmarks being established [16]. A strong point in our case report is the use of the cutting guide, designed for the specific patient. It was planned to fix the guide to the bone by means of four 2.0 mm Steinmann pins. The holes of the pins of the guide correspond with the holes for the screws of the prothesis, in order to have a perfect spatial positioning of the PGR consistent with the pre-operative planning and without risk of recurrent patellar luxation or malalignment.

In this case report, the use of the custom-made implant became indispensable for the treatment of severe bone lesions secondary to osteomyelitis. Osteomyelitis is an infectious condition that affects the bone, causing progressive inflammation which leads to the destruction of bone tissue and the formation of new bone [1,10]. The management of osteomyelitis entails the administration of a prolonged antibiotic therapy and the debridement or resection of infected and necrotic bone, depending on the severity and extent of the infectious process [6,11]. This therapeutic approach aims to eradicate the infected tissue and facilitate osseous healing [6,11]. In our patient, a persisting bone sequestrum perpetuated the infection, manifesting as recurrent fever that exhibited only partial responsiveness to systemic antibiotic therapy. A month had elapsed between the onset of osteomyelitis and bone sequestrum removal, and this period of latency was responsible for severe articular impairment characterized by trochlear cartilage and subchondral bone destruction. The excision of the sequestrum, followed by targeted antibiotic therapy, effectively eradicated the infection both locally and systemically. Subsequently, after one month, grade IV lameness persisted in the affected limb, but the absence of systemic symptoms indicative of infection, such as hyperthermia, lethargy and inappetence, was observed in the patient. Considering the young age of our patient, the anamnesis of endocarditis and absence of a traumatic event, and the metaphyseal location of the osteomyelitis, there was a strong suspicion of hematogenous osteomyelitis [1,6]. In human medicine, the incidence of vertebral osteomyelitis is high in patients with infective endocarditis [40], but this correlation between osteomyelitis and endocarditis was not revealed in veterinary medicine.

Staphylococcus spp. is the predominant bacteria detected in approximately 60% of osteomyelitis cases in dogs and cats, along with Escherichia coli and Streptococcus spp. Additionally, other Gram-negative microorganisms such as Pasteurella, Pseudomonas, Proteus, Serratia and Klebsiella species, as well as Gram-positive organisms like Corynebacterium spp. and enterococci, have also been isolated [1,3,41]. In our patient, Klebsiella oxytoca was isolated in the synovial liquid and in the adjacent bone tissue. It is not a single species but a complex comprising at least six species, i.e., Klebsiella grimontii, Klebsiella huaxiensis, Klebsiella michiganensis, K. oxytoca, Klebsiella pasteurii and Klebsiella spallanzanii [42]. In humans, K. oxytoca is a member of the normal gut microflora but is also an important human pathogen causing a large variety of infections ranging from mild diarrhea to life-threatening bacteremia, meningitis [42,43] and endocarditis [44]. In the literature, there is a paucity of reports that describe Klebsiella oxytoca infection in dogs [45].

The limitations of this report include the fact that it was based on a single case, even though it showed excellent short-, medium- and long-term outcomes [24]. Furthermore, postoperative complications such as aseptic loosening and mechanical failure of the implant may not become evident during follow-up until 18 months after the surgery.

## 4. Conclusions

To the best of our knowledge, this is the first report which describes the use of a custom-made PGR prothesis. Based on the clinical, radiographic and tomographic outcomes, the use of a custom-made PGR prothesis was an effective treatment to restore the articular surface of the femoral trochlea and resolve the bone defect.

## Data Availability

The data present in this study are available within the article.

## Supplementary Materials

The following supporting information can be downloaded at: https://www.mdpi.com/article/10.3390/ani14060909/s1. Video S1: The patient manifested lethargy, inappetence and anorexia, and no-weightbearing lameness and severe edema of the right hind limb were revealed. Video S2: At 12 months of age, the patient manifested grade III lameness, significant muscle hypotrophy, pain on manipulation of the right stifle, joint swelling and grade III medial patellar luxation. The severe edema of the limb observed during the first orthopedic evaluation disappeared. Video S3: (a) At one-day postoperative follow-up, the patient revealed grade III right limb lameness [19]. During the following follow-up at (b) 3 months and (c) 18 months, the lameness of the right limb decreased from grade II to grade 0 [19], respectively.
